# Descriptive and network analyses of the equine contact network at an equestrian show in Ontario, Canada and implications for disease spread

**DOI:** 10.1186/s12917-017-1103-7

**Published:** 2017-06-21

**Authors:** Kelsey L. Spence, Terri L. O’Sullivan, Zvonimir Poljak, Amy L. Greer

**Affiliations:** Department of Population Medicine, Ontario Veterinary College, University of Guelph, Guelph, ON N1G 2W1 Canada

**Keywords:** Biosecurity, Equine, Infectious disease, Social network analysis

## Abstract

**Background:**

Identifying the contact structure within a population of horses attending a competition is an important element towards understanding the potential for the spread of equine pathogens as the horses subsequently travel from location to location. However, there is limited information in Ontario, Canada to quantify contact patterns of horses. The objective of this study was to describe the network of potential contacts associated with an equestrian show to determine how this network structure may influence potential disease transmission.

**Results:**

This was a descriptive study of horses attending an equestrian show in southern Ontario, Canada on July 6 and 7, 2014. Horse show participants completed a questionnaire about their horse, travel patterns, and infection control practices. Questionnaire responses were received from horse owners of 79.7% (55/69) of the horses attending the show. Owners reported that horses attending the show were vaccinated for diseases such as rabies, equine influenza, and equine herpesvirus. Owners demonstrated high compliance with most infection control practices by reporting reduced opportunities for direct and indirect contact while away from home. The two-mode undirected network consisted of 820 nodes (41 locations and 779 horses). Eight percent of nodes in the network represented horses attending the show, 87% of nodes represented horses not attending the show, but boarded at individual home facilities, and 5% represented locations. The median degree of a horse in the network was 33 (range: 1–105).

**Conclusions:**

Developing disease management strategies without the explicit consideration of horses boarded at individual home facilities would underestimate the connectivity of horses in the population. The results of this study provides information that can be used by equestrian show organizers to configure event management in such a way that can limit the extent of potential disease spread.

## Background

The globally expanding livestock industry has made the prevention and control of infectious diseases more challenging. Animal movements have been internationally recognized as a risk factor for disease introduction and spread, notably following outbreaks such as the 2001 foot-and-mouth disease outbreak in the United Kingdom [1] and the 2007 equine influenza outbreak in Australia [2]. Opportunities for the introduction and spread of disease exist as animals move between locations due to the potential for contact with animals outside of their routine daily contacts (i.e. usual barn mates). An understanding of animal movement and contact patterns is essential to identify the risk of disease spread within a population and to determine intervention strategies in the event of an outbreak. ﻿The Canadian equine industry contributed more than $19 billion to the Canadian economy in 2010 [3]. Horses within the industry are highly mobile, travelling locally, regionally, and nationally to participate in show and sporting events. Previous studies have characterized animal contact patterns at these types of shows, including a network of sheep attending agricultural shows [4] and a network of donkeys attending equestrian shows [5]. In recent years, disease outbreaks at equestrian shows have become more prevalent. Examples include the introduction and widespread transmission of equine influenza in Australia in 2007, which was thought to occur after the importation of an infected horse for a competition [6, 7], and an outbreak of equine herpesvirus-1 in the USA in 2011, which occurred after horses gathered at a competitive event [8].

Epidemiological approaches that do not explicitly consider contact patterns may be insufficient to estimate the risk of disease spread within the equine population [9–11]. Social network analysis is an approach that is generally used to explore and characterize the relationship between a group of individuals or locations [10]. In veterinary epidemiology, social network analysis has been previously used to characterize livestock contact and movement patterns to estimate the potential risk of disease spread [9, 11–14].

In some countries, the availability of a national database has allowed for full characterization of equine movement and contact networks [11]. However, the current ability to trace equine contacts and travel patterns in Ontario is limited and there is substantial variability in individual-level record keeping regarding these travel patterns [3]. The objective of this study was to describe the network of potential contacts associated with a single equestrian show in southern Ontario to determine how network structure may affect potential disease transmission.

## Methods

### Study location

This was a descriptive study of horses attending an equestrian show in Orangeville, Ontario, Canada on July 6 and 7, 2014. The equestrian show of interest was an Equine Canada sanctioned 2-day silver level dressage competition [15]. Upon entering the competition, participating riders could choose to have their horse stabled on-site for the duration of the show. The fairgrounds had two unique locations where participating horses could be stabled: the main barn and the coverall barn. Other participating horses were trailered in daily (ship-ins), meaning they did not stable at the facility overnight, but rather travelled to the fairgrounds and remained in a separate outdoor location (field parking area) until their warm up and/or competition time. For the purpose of this study, this location was referred to as the field.

### Questionnaire and data collection

A questionnaire was developed using a three-stage process, which included design, pre-testing using personal interviews, and final distribution. For this study, the home facility was defined as the facility where a horse spent the majority of its time. The questionnaire contained 18 questions relating to 1) information about the competing horse (age, sex, stabling location, and vaccination status); 2) information about the horse’s home facility (geographic location, total number of horses boarded, number of owners that boarded horses at the facility, and presence of breeding mares, foals, and senior horses at the facility); 3) average number of incoming and outgoing horse movements from the home facility per month; 4) travel patterns of the horse 6 months prior to the show and 6 months following the show; and 5) the opportunities for contact to occur both at the home facility and while stabled away from home (direct nose-to-nose contact, sharing equipment, sharing cleaning tools, sharing water/feed, and sharing a wash rack).

Participants were made aware of the research project and data collection activities prior to the show date through advertisement on the social media pages of the equestrian organization. During the show, periodic announcements over the speaker and personal interactions with show participants aimed to increase awareness of the study. Owners, trainers, and/or riders were asked to complete the 2-page questionnaire on-site regarding the horse that they were responsible for at the show. Participants were eligible to complete the questionnaire if they were 18 years of age or older, had a horse present at the show, and were registered to compete. Only one questionnaire per horse was accepted, and duplicates were avoided by asking for the unique competition entry number of the horse. Participants could complete a questionnaire for more than one horse if they were responsible for several horses. Participants submitted their completed questionnaire in a locked box on-site, which was kept closed until the research team left the fairgrounds. Questionnaires were anonymous as no personally identifying information was collected. Information on the horses of non-responding owners, such as their stabling location, age, and sex, was collected from publically available competition entry data on the equestrian organization’s website.

The statistical software package Stata (StataCorp. 2013. Stata Statistical Software: Release 14. College Station, TX: StataCorp LP) was used for all descriptive analyses. Statistically significant differences (*P*-value <0.05) were assessed between the proportion of participants that reported opportunities for contact at the home facility compared to opportunities for contact away from home using the Fisher’s exact test. Spearman’s correlation coefficient and the Wilcoxon rank-sum test were used to evaluate statistically significant differences between the estimated number of horse movements 6 months prior to the show and 6 months following the show by age and sex.

### Network analysis

A two-mode undirected network was created to represent the relationships between horses and locations. The first mode consisted of individual horses, which included horses that were competing at the show (primary contacts) and horses stabled at the home facilities of those competing horses (secondary contacts). The second mode consisted of locations, which included areas at the fairgrounds and individual home facilities. Edges between a horse and a location represented the presence of the horse in that location. Horses were categorized by location through a question on the questionnaire that asked about its boarding location at the fairgrounds. Some horses at the fairgrounds boarded at the same home facility, and therefore multiple horses at the show could be connected to one home facility. Horses that attended the show but had owners that did not complete the questionnaire were included in the network, but were not connected to any home facility.

The two-mode network was projected as a one-mode network by creating a horse-by-location matrix and multiplying this matrix by its transpose. When plotted as a graph, the one-mode network provides a visual representation of the connections between individual horses based on co-boarding at the same locations, assuming that all horses in that location have the same potential for contact. By projecting as a one-mode network, the connections between individual horses could be described by calculating network measures. Edges in the one-mode network were unweighted, as information on the intensity and duration of horse-to-horse contact within each location was not collected. The networks were visualized using Gephi v. 0.8.1-beta [16].

Descriptive network measures for the one-mode network were calculated in the statistical software R using the ‘igraph’ library [17]. These measures included: density, which is the proportion of connections among horses in the network relative to the total number of possible connections [10]; diameter, which is the largest geodesic distance between any two horses in the network [18]; path length, which is the number of distinct steps between any two horses [18]; and clustering coefficient, which measures the proportion of horse connections that are also connected to one another [10].

Measures of centrality were also calculated to provide an indication of the importance of a given horse based on how connected it is in the network [10]. In an undirected network, the degree is the number of connections of a horse [10]. Betweenness centrality estimates how often a horse is found on the shortest path between any two horses [18]. A higher betweenness score is assigned if the horse indirectly connects many other horse pairs. Closeness centrality estimates how closely connected a horse is to other horses in the network [18]. A high normalized closeness centrality score reflects a horse that has a short path distance to every other horse, thus being indirectly or directly reachable by other horses in the network [19]. The Eigenvector centrality score measures the importance of a horse in the network by assigning its score relative to its connections to others, so that high-scoring neighbours of a horse will contribute more to its individual score [19].

## Results

### Horse demographics

Questionnaire responses were received from horse owners of 55 out of the 69 horses attending the show (response rate: 79.7%). The average age of a horse at the show was 9.6 years (range 4–24 years). The majority of horses attending the show were geldings (33/55), followed by mares (21/55), and one stallion. Sixty-four percent (35/55) of participants had horses that were stabled overnight at the event; of those participants, 43% (15/35) were stabled in the barn and 57% (20/35) in the coverall. Sixty-nine percent (37/54) of participants stated there were other horses from their home facility participating at the same show. Of those participants, 73% (27/37) stated that these horses were stabled in neighbouring stalls, while 16% (6/37) did not provide information on the boarding location of the other horses.

### Home facilities

**Fig. 1:**
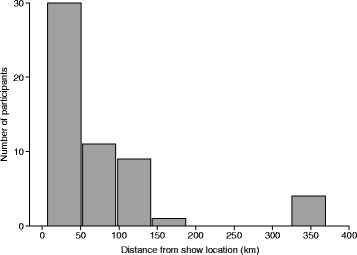
Geographic distance between participants’ home facilities and the equestrian show location

Descriptions of participants’ home facilities are presented in Table 1. The home facilities of 55% of participants (30/55) were located less than 50 km away from the show location (range 6–370 km) (Fig. 1). The majority of participants (80%) came from home facilities that housed horses owned by four or more owners. The average number of horses boarded at individual home facilities was 22 (median = 12, range 2–85).

**Table 1:** Descriptive home facility characteristics, obtained from questionnaires collected at an equestrian show in Ontario, Canada

Characteristic		Proportion	Percent (%)
Number of owners with horses at facility	1	7/55	12.7
	2–3	4/55	7.3
	4+	44/55	80.0
Types of horses at facility^a, b^	Breeding mares	17/54	31.4
	Foals	15/54	27.8
	Seniors	43/54	79.6
Number of resident horses transported on/off facility per year	1–5	33/55	60.0
	6–10	9/55	16.4
	11–15	6/55	10.9
	16–20	7/55	12.7

^a^The number of participants that indicated that type of horse resided at the facility. Participants could choose more than one category to describe the horses at their home facility

^b^One participant did not provide a response to this question on the questionnaire

### Horse movements

Descriptions of horse movements from participants’ home facilities are presented in Table 2. The estimated number of horses that entered a participant’s home facility per year (for reasons such as training, shows, new boarders, breeding, etc.) ranged from 0 to 500 (median = 4). There were no statistically differences between the median number of times a horse travelled relative to the show date depending on its sex (6-month period prior to the show date: mares and geldings, z = −0.62, *P*-value = 0.53; mares and stallions, z = 0.76, *P*-value = 0.45; geldings and stallions, z = 0.46, *P*-value = 0.64. Six-month period following the show date: mares and geldings, z = 0.78, *P*-value = 0.43; mares and stallions, z = 0.25, *P*-value = 0.80; geldings and stallions, z = 0.56, *P*-value = 0.57.). In addition, there was no significant correlation between the number of times a horse travelled relative to the show date and its age (6-month period prior to the show date: rho = 0.22, *P*-value = 0.10; 6-month period following the show date: rho = −0.13, *P*-value = 0.37).

**Table 2:** Owner-reported horse movements from home facilities, collected at an equestrian show in Ontario, Canada

Characteristic		Mean	Median	Range	
				Min.	Max.
Number of boarding horses at home facility		22	12	2	85
Number of new incoming horses per year		35.1	3.5	0	500
Off-site trips relative to show date	Past 6 months	5.6	3.5	0	36
	Next 6 months^a^	9.2	4	1	75
Overnights at another facility in past 12 months		11.5	2	0	120

^a^Based on participants’ estimate of how many trips were planned during this time period

### Infection control and biosecurity

**Fig. 2:**
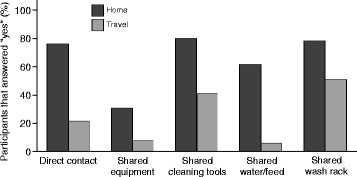
Type of horse-to-horse contact occurring at home facility and while travelling. Percentage of participants that stated there were opportunities for contact between horses at their home facility (*dark grey bars*) and while travelling away from home (*light grey bars*). Types of contact included ways in which infectious diseases could potentially be transmitted

Individuals reported a reduction in the opportunities for contact to occur between horses while away from their home facility (Fig. 2). However, there were no statistically significant differences between the proportion of participants that reported opportunities for contact at the home facility compared to the proportion of participants that reported opportunities for contact away from home (direct nose-to-nose contact, *P*-value = 0.25; sharing equipment, *P*-value = 0.10; sharing cleaning tools, *P*-value = 0.49; sharing water/feed, *P*-value = 1.00; sharing a wash rack, *P*-value = 1.00). While most participants indicated that direct nose-to-nose contact of horses occurred both at their home facility and while away from home (76 and 22%, respectively), participants reported a reduction in sharing equipment, cleaning tools, and water/feed when they travelled away from home. Seven percent of participants indicated that there were no types of contact that occurred at the home facility, compared to 33% of participants that stated no types of contact occurred while away from home.

Owner-reported vaccine coverage levels in the past 12 months included equine influenza virus (96%, 50/52), rabies (90%, 47/52), strangles (60%, 31/52), West Nile virus (88%, 46/52), eastern equine encephalitis and western equine encephalitis (85%, 44/52), equine herpesvirus (73%, 38/52), and tetanus (83%, 43/52). Only one participant stated that their horse was unvaccinated for all diseases listed, while 7% (4/54) were unsure of the vaccination status of their horse.

### Network analysis

**Fig. 3:**
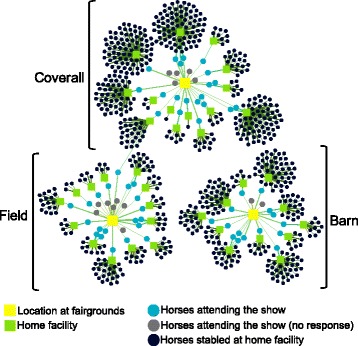
Two-mode network of primary and secondary horse contacts. The square nodes represent locations and the circle nodes represent horses. An edge represents a connection between a horse and a location

**Fig. 4:**
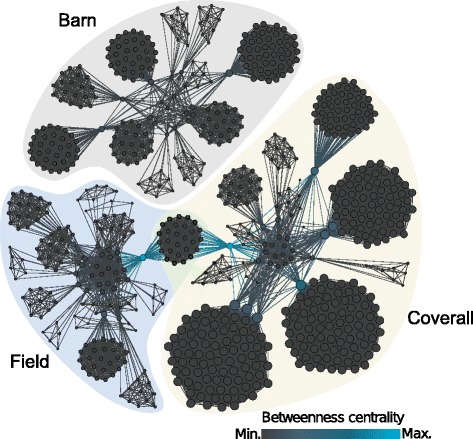
Horse contacts projected as a one-mode network. All nodes represent horses, and edges represent connections facilitated through being in a common location. Node colours represent betweenness centrality scores. The size of the node represents degree, where larger nodes have a higher degree and smaller nodes have a lower degree

The two-mode network of horses attending the show consisted of 820 nodes (41 locations and 779 horses) and 834 edges (Fig. 3). Five percent of nodes in the network represented locations: 4.6% (38/820) represented individual home facilities and 0.4% (3/820) represented the separate stabling or ship-in locations at the fairgrounds. Only 8% (69/820) of the nodes in the network represented horses that were competing at the show, while 87% (710/820) represented horses stabled at individual home facilities. When the two-mode network was projected as a one-mode network, there were 779 nodes (horses) and 16,032 edges (Fig. 4). Network measures calculated from the one-mode network are listed in Table 3.

**Table 3:** Descriptive measures of the one-mode network of horses attending a single equestrian show in Ontario, Canada

Network measure	Number	Median	Range	
			Min.	Max.
Nodes	779	--	--	--
Edges	16,032	--	--	--
Density	0.05	--	--	--
Diameter	5	--	--	--
Clustering coefficient	0.97	--	--	--
Average path length	3.28	--	--	--
Degree	--	33	1	105
Betweenness centrality	--	0	0	65,156.18
Closeness centrality^a^	--	0.0042	0.0018	0.0042
Eigenvector centrality	--	0.000068	0	0.11

^a^Calculated as normalized closeness centrality

The median (range) degree of the nodes in the one-mode network was 33 (1–105). The nodes with the smallest degree were secondary contacts at two separate home facilities where only those horses and the competing horses were boarded. The nodes with the highest degree corresponded to three competing horses that boarded at the same home facility, which housed 80 horses. The same three nodes also had the highest Eigenvector centrality scores. The node with the highest betweenness centrality and closeness centrality scores was a horse that was stabled in the coverall, but boarded at the same home facility as another competing horse that was shipped-in and remained in the field.

## Discussion

This study has provided a description of horses and home facilities related to a single equestrian show in southern Ontario, Canada in July 2014. This study has also described the network of potential contacts associated with this show. The findings presented in this study contribute to a better understanding of the contact patterns of horses attending an equestrian show. The inclusion of the secondary contacts in the network demonstrated the high amount of connectivity beyond the horses that were present at the show, highlighting the importance of describing these contacts when estimating the risk of disease spread in the population.

The sampling method for this study was a convenience sample of horse owners/trainers/riders at the show, and therefore may not be representative of the general Ontario equine population. However, the high response rate for the questionnaire suggests that the network is fairly well characterized for horses associated with this particular show. Previous contact networks in veterinary medicine have been constructed using databases of animal movements [11, 20] or information obtained through registries [21], however, such information is not available in Ontario. Simply using a complete list of registrants at the show would not have allowed for the detailed collection of data about individual home facilities, or the identification of secondary contacts at these home facilities.

Most horses were boarded at home facilities less than 50 km away from the fairgrounds, suggesting that potential disease spread initiating at the show would have a higher probability of being contained in the local area due to the majority of contacts residing in close geographic proximity. Since only a small proportion of horses were boarded at locations farther away from the show location, wide geographic spread of a potential disease via horses travelling back to their facilities would be less likely. The majority of horses residing in close geographic proximity to the show location could be explained by the equestrian sport of interest (dressage) and the type (silver level competition) of equestrian show being studied. In Canada, dressage has three competition levels that relate to the type of membership purchased: bronze, silver, and gold. Each level may attract a different group of competitors based on the competitiveness of their horse and if they wish to compete locally (bronze), provincially (silver), or nationally (gold) [15]. Differences in the contact network structure could be expected between different competition levels or equestrian sports. For instance, a network of horses that exclusively competed at the gold level might have more contacts over a wide geographic range. The potential difference in network structure between competition levels and equestrian sports is an area identified for future research.

The horses in this study were vaccinated for most equine diseases, and had an owner-reported vaccine coverage level that was much higher than previously reported for Ontario horses [22]. The differences in vaccination coverage reported previously for Ontario horses may be attributed to the differences in study populations. The population of the previous study was involved in an investigation of respiratory disease outbreaks in Ontario, which may suggest why the proportion of horses that were vaccinated prior to the outbreaks was low [22]. Alternatively, the horses in the current study may be highly vaccinated due to their frequent participation in equestrian events. Although vaccination is not required to participate in most shows in Ontario, it is recommended practice for horses that travel frequently [23].

The absence of a statistically significant difference between opportunities for contact at the home facility and while away from home could be due to the small sample size in this study. Regardless, questionnaire responses indicated that participants reported decreased horse-to-horse contact when travelling away from home. This indicated that the potential for disease transmission while travelling away from home may be reduced due to an owner’s awareness of good biosecurity practices. These results might be an overestimate due to obsequiousness bias, where participants could have responded with what they deemed would be an acceptable answer (i.e. that they vaccinate their horse because it is recommended practice, even if they do not). Steps to minimize this bias were taken by emphasizing the anonymous nature of the questionnaire and through the use of the locked questionnaire submission box.

The majority of horses in the one-mode network were secondary contacts, demonstrating the high amount of connectivity beyond the primary horses that attended the show. Simply planning disease intervention strategies based on the horses that attended the show without explicit consideration of secondary contacts would severely underestimate the resources required to control a potential outbreak. The quantification of contacts at an equestrian show can aid in developing disease management plans in the event of a future inadvertent introduction of an equine disease. In addition, visualizing the contact network associated with an equestrian show can act as an education tool to demonstrate the importance of practicing good biosecurity behaviours.

The low density of the network indicates that the likelihood of an infectious disease spreading to every horse in the network by direct contact is low. However, the impact of this effect is difficult to measure without the consideration of incoming and outgoing infection chains, which can only be measured in directed networks while considering the chronological order of the contacts [24]. The high clustering coefficient was likely due to the naturally clustered nature of equine facilities, as horses in the same location had direct connections with one another, creating multiple clusters of horses. The high clustering coefficient might indicate that a highly infectious disease could potentially spread quickly within a single facility.

The two horses with the highest betweenness and closeness centrality scores had contact with horses in three locations, indicating that they were the most important horses for potential disease spread in the network. In terms of disease transmission, centrality measures can indicate influential nodes in the network; betweenness centrality can indicate gatekeepers for transmission, and closeness centrality indicates a horse that is a short distance from most others, so a disease from a random horse in the network could potentially reach the central horse quickly [10, 18]. The two horses with the highest scores acted as cutpoints between two separate locations at the fairgrounds, allowing horses in these locations to be connected in the network. This type of information could be useful during the design/planning stages of boarding locations at equestrian shows. If these two horses had co-boarded at the same location at the fairgrounds, the network would consist of three separate components, which would lead to a reduced risk of disease since horses from each component would not be reachable from the others.

Nodes with high betweenness centrality values but low Eigenvector values can act as important gatekeepers for disease transmission because they connect otherwise isolated horses to the central core of the network [13]. The horse with these corresponding scores was the only horse from its home facility that participated in the show, which means that potential disease spread to/from the home facility could only occur through that horse. Nodes with low betweenness scores but high Eigenvector values have direct contact to important nodes in the network [13]. The two horses with these corresponding scores were neighbours to the horses that acted as the central connecting nodes between the coverall barn and the field.

Limitations of this study include the potential for recall bias, as participants completed the questionnaire on-site and did not have access to their horse’s records to answer questions regarding their previous travel patterns and vaccination status. Some questions were designed to minimize recall bias by providing categories for participants to select an answer (i.e. questions about the number of owners per facility and the average range of horses transported on/off the facility per month). Individuals that travelled with their horse more often might have been less precise in their estimate of the number of times their horse travelled in the 6 months prior to the show date, while those that travelled less often might have been more likely to recall the number of times that they had travelled. Limitations of the network analyses include the static nature of the network, as this does not consider the effect of changing contact structure as horses move in and out of the home facility. It is important to note that the static nature of the network means that the network measures calculated in this study may not persist beyond the study period. In addition, the network structure and characteristics may change if movements beyond this competition were incorporated. Further research should explore the effect of ongoing movements within the equine population on the network structure and potential disease dynamics.

Previous equine contact networks have used a variety of definitions for connections between horses and locations, including connections between racehorse trainers while racing together [20] and connections between equine facilities as horses moved between them [11]. Co-attending the same competitions has been used previously in the UK sheep population as a proxy for a connection in network analysis [21]. In the absence of detailed data on direct contacts within facilities, the definition of a contact in this current study may be an oversimplification because it assumes that all horses in the same location have the same probability of contacting one another. Additionally, the definition of a contact between horses may depend on the specific disease of interest. For example, an assumption that horses are in contact with one another at the same location may be reasonable for respiratory diseases such as equine influenza, which can be transmitted via airborne respiratory droplets [25]. Further investigations are required to determine the frequency and intensity of direct contact between horses co-boarding in the same location.

## Conclusion

To the authors’ knowledge, this study provides the first description of an equine contact network in Ontario, Canada. Questionnaire responses indicated that horses attending the show were vaccinated for diseases such as rabies, equine influenza, and equine herpesvirus, and participants acted preventatively by reducing opportunities for direct and indirect contact while travelling away from home. The contact structure described in this study can be used to determine effective disease prevention and control strategies to reduce the risk of future outbreaks in this population.

## References

[1] Ortiz-Pelaez A, Pfeiffer DU, Soares-Magalhães RJ, Guitian FJ (2006). Use of social network analysis to characterize the pattern of animal movements in the initial phases of the 2001 foot and mouth disease (FMD) epidemic in the UK. Prev Vet Med.

[2] Bell IG, Drury-Klein C (2011). Analysis of horse movements and events during the 2007 outbreak of equine influenza in New South Wales, Australia. Aust Vet J.

[3] Equine Canada (2011). Canadian Horse Industry Profile Study.

[4] Webb CR (2006). Investigating the potential spread of infectious diseases of sheep via agricultural shows in Great Britain. Epidemiol Infect.

[5] Finney S, Collins JA, Duggan V (2015). An investigation of the equine infectious disease threat represented by the presence of donkeys at mixed equestrian events in Ireland. Ir Vet J.

[6] Kirkland P, Davis R, Wong D, Ryan D, Hart K, Corney B, Hewitson G, Cooper K, Biddle A, Eastwood S, Slattery S, Rayward D, Evers M, Wright T, Halpin K, Selleck P, Watson J (2011). The first five days: field and laboratory investigations during the early stages of the equine influenza outbreak in Australia, 2007. Aust Vet J.

[7] Moloney B (2011). Overview of the epidemiology of equine influenza in the Australian outbreak. Aust Vet J.

[8] Traub-Dargatz JL, Pelzel-Mccluskey AM, Creekmore LH, Geiser-Novotny S, Kasari TR, Wiedenheft AM, Bush EJ, Bjork KE (2013). Case-control study of a multistate equine herpesvirus myeloencephalopathy outbreak. J Vet Intern Med.

[9] Firestone SM, Ward MP, Christley RM, Dhand NK (2011). The importance of location in contact networks: Describing early epidemic spread using spatial social network analysis. Prev Vet Med.

[10] Dubé C, Ribble C, Kelton D, McNab B (2009). A review of network analysis terminology and its application to foot-and-mouth disease modelling and policy development. Transbound Emerg Dis.

[11] Sánchez-Matamoros A, Martínez-López B, Sánchez-Vizcaíno F, Sánchez-Vizcaíno JM (2013). Social network analysis of Equidae movements and its application to risk-based surveillance and to control of spread of potential Equidae diseases. Transbound Emerg Dis.

[12] Dubé C, Ribble C, Kelton D (2010). An analysis of the movement of dairy cattle through 2 large livestock markets in the province of Ontario, Canada. Can Vet J.

[13] Dorjee S, Revie CW, Poljak Z, McNab WB, Sanchez J (2013). Network analysis of swine shipments in Ontario, Canada, to support disease spread modelling and risk-based disease management. Prev Vet Med.

[14] Hayama Y, Kobayashi S, Nishida T, Muroga N, Tsutsui T (2012). Network simulation modeling of equine infectious anemia in the non-racehorse population in Japan. Prev Vet Med.

[15] Equestrian Canada. https://www.equestrian.ca. Accessed 29 Jul 2016.

[16] Bastian M, Heymann S, Jacomy M (2009). Gephi: an open source software for exploring and manipulating networks. International AAAI Conference on Weblogs and Social Media.

[17] Csardi G, Nepusz T. The igraph software package for complex network research. InterJournal, Complex Syst. 2006:1695.

[18] Martínez-López B, Perez AM, Sánchez-Vizcaíno JM (2009). Social network analysis. Review of general concepts and use in preventive veterinary medicine. Transbound Emerg Dis.

[19] Borgatti SP, Everett MG, Johnson JC (2013). Analyzing Social Networks.

[20] Christley RM, French NP (2003). Small-world topology of UK racing: the potential for rapid spread of infectious agents. Equine Vet J.

[21] Webb CR (2005). Farm animal networks: unraveling the contact structure of the British sheep population. Prev Vet Med.

[22] Diaz-Mendez A, Viel L, Hewson J, Doig P, Carman S, Chambers T, Tiwari A, Dewey C (2010). Surveillance of equine respiratory viruses in Ontario. Can J Vet Res.

[23] Guidelines for the vaccination of horses. http://www.omafra.gov.on.ca/english/livestock/horses/facts/info_vaccine.htm. Accessed 25 Apr 2016.

[24] Büttner K, Krieter J, Traulsen I (2013). Characterization of contact structures for the spread of infectious diseases in a pork supply chain in Northern Germany by dynamic network analysis of yearly and monthly networks. Transbound Emerg Dis.

[25] Timoney PJ (1996). Equine influenza. Comp Immunol Microbiol Infect Dis.

